# The mechanism of 45S5 bioactive glass-mediated, cell-type-specific death of bone tumor cells

**DOI:** 10.1038/s41420-026-03211-x

**Published:** 2026-07-01

**Authors:** Joerg Fellenberg, Sarina Losch, Marcela Arango-Ospina, Fabian Westhauser, Burkhard Lehner, Aldo R. Boccaccini

**Affiliations:** 1 Research Centre for Molecular and Regenerative Orthopaedics, Department of Orthopaedics, Heidelberg University Hospital, Heidelberg, Germany; 2 Institute of Biomaterials, Department of Materials Science and Engineering, University of Erlangen-Nuremberg, Erlangen, Germany; 3 Department of Orthopaedics, University of Regensburg, Bad Abbach, Germany

**Keywords:** Bone cancer, Stress signalling

## Abstract

Despite advances in treatment modalities, current bone tumor therapies still face significant challenges, including severe side effects of conventional approaches, poor survival rates and high rates of tumor recurrence. We previously identified a strong cytotoxic effect of 45S5-bioactive glass (BG) particles on bone tumor cells, indicating possible therapeutic properties of these materials. In order to identify the mechanisms that trigger tumor cell death, we investigated the impact of 45S5-BG on bone tumor cells compared to non-neoplastic cells. Regardless of cell type, BG treatment induced oxidative stress and increased ROS production. Fluorescently labeled SiO_2_ particles were rapidly internalized by both cell types. Tumor cell-specific responses included elevation of calcium levels, activation of MAP- and AKT-kinase as well as the release of LDH and ATP from the cells. Apoptosis, pyroptosis and necroptosis could be excluded as possible cell death mechanisms due to the lack of caspase activation and a RIPK3 deficiency of the tumor cells. Rather, we identified an important role of lysosomes and the requirement of intact V-ATPase activity. Together with a strong increase in intracellular Fe^2+^ and MDA levels triggered by BG-treatment, these data strongly suggest that ferroptosis is the main cause of tumor cell-specific death. In combination with the known beneficial effects of BGs on bone regeneration, our data point to a potential therapeutic strategy for promoting bone repair while simultaneously reducing tumor recurrence. Furthermore, understanding the underlying mechanisms and the option to modify the glass composition could help to develop even more potent BG variants.

## Introduction

Bioactive glasses (BG) are a class of surface-reactive inorganic biomaterials, with exceptional biocompatible and bioactive properties ideally suited for repair and replacement of damaged or diseased bone structures. The most widely studied and clinically established bioactive glass is the 45S5 composition, originally developed by Larry Hench and colleagues in 1969. This formulation consists of 45 wt% silicon dioxide (SiO₂), 24.5 wt% calcium oxide (CaO), 24.5 wt% sodium oxide (Na₂O), and 6 wt% phosphorus pentoxide (P₂O₅). The unique distinction of BGs lies in their ability to interact dynamically with biological fluids and tissues. Unlike biologically inert materials such as aluminum oxide or titanium, BGs actively degrade in body fluids while releasing therapeutic ionic products, including silicon, calcium, phosphate ions and many more, depending on the BG composition [1]. This property enables simultaneous tissue regeneration and controlled biomaterial resorption, allowing the material to achieve intimate physicochemical bonding with natural bone tissue through the formation of a hydroxyapatite-like mineral layer at the glass-tissue interface [2–4]. Furthermore, the capacity to modify the chemical composition of BGs enables the targeted modulation of their ion release profile. This renders them a versatile material for a wide range of medical applications [5]. In clinical practice, BGs have been established for the treatment of various bone defects arising from trauma, benign or malignant tumors, or congenital diseases. In addition to their regenerative potential, BGs exhibit antimicrobial and angiogenic properties that provide supplementary clinical benefits [4, 6]. Recent studies have explored the potential of BGs for tumor therapy applications [3]. One innovative approach exploits mesoporous BGs as drug delivery systems due to their enlarged surface area and high porosity, enabling loading with cytostatic agents for controlled, targeted drug release directly to tumor cells [7]. Further applications include the use of magnetic BGs for the induction of hyperthermia [8], or doping BGs with therapeutic ions like iron, lithium or gallium [9].

In addition, preliminary evidence suggests that BGs may also exert direct cytotoxic effects on tumor cells without requiring additional pharmaceutical agents. Internalization of nano- and micro-sized BG-particles has been demonstrated in osteoblasts where they displayed a size-dependent mechanism of intracellular localization and cytotoxicity [10]. A dose-dependent cytotoxic influence of BG particles has further been shown in fibroblasts, pre-osteoblasts and osteosarcoma cells [11]. Recent studies have identified selective cytotoxic effects of BGs against tumor cells while sparing non-neoplastic cells. In contrast to normal human skin fibroblasts, non-small cell lung cancer cells respond to BG treatment with enhanced production of reactive oxygen species (ROS), resulting in substantial loss of DNA integrity and collapse of mitochondrial membrane potential [12]. In a previous study, we demonstrated cytotoxic effects of BGs on neoplastic stromal cells derived from giant cell tumors of bone, while mesenchymal stromal cells survived when exposed to identical conditions [13]. A selective toxicity of BGs toward tumor cells while preserving or even enhancing function of non-neoplastic cells represents a unique therapeutic advantage. Such dual functionality - simultaneous bone regeneration and tumor cell suppression - creates novel opportunities for integrated treatment approaches in bone oncology, where both, the repair of large bone defects and the prevention of tumor recurrence need to be addressed. Despite accumulating evidence for selective cytotoxic effects of BGs on tumor cells, the underlying mechanisms remain poorly characterized. A comprehensive understanding of these mechanisms is imperative in order to facilitate the design of potent BG variants, which will enable the development and optimization of new bone tumor therapies that are urgently needed. The present study aimed to investigate the cytotoxic effects of 45S5-BG on different bone tumor cell lines in direct comparison with non-neoplastic bone cells. The study elucidates the molecular and cellular mechanisms driving selective toxicity, and evaluates the potential therapeutic applications of these findings for integrated bone tumor treatments.

## Results

### Tumor cell-specific cytotoxicity of 45S5-BG particles

**Fig. 1:**
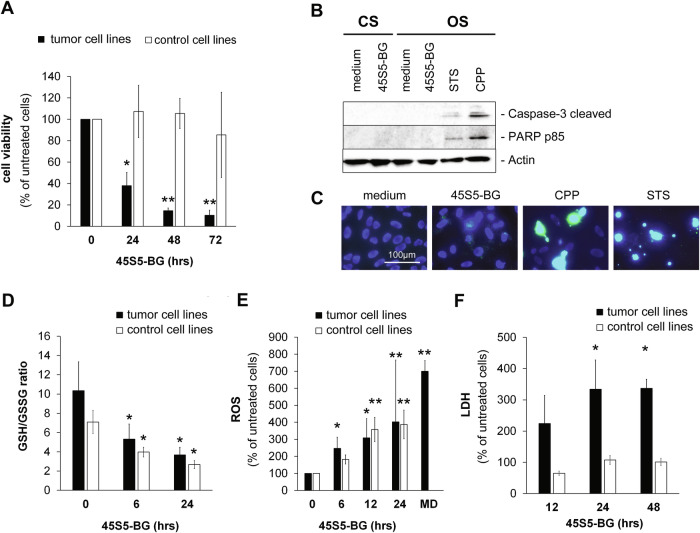
45S5-BG particles cause oxidative stress and induce caspase-independent cell death exclusively in tumor cells. **A** Tumor cell lines and control cell lines (*n* = 4 each) were cultured with 45S5-BG particles ( < 25 µm, 0.5 mg/ml) for the indicated time before cell viability was measured by WST-1 assay. **B** Western blot analysis of cleaved caspase-3 and the PARP p85 in tumor cells with and without 45S5-BG treatment for 24 h. The known apoptosis inducers Staurosporine (STS, 200 nM) and Cisplatin (CPP, 5 µg/ml) were used as positive controls. Actin served as loading control. **C** Detection of apoptotic cells by NucView488 staining (green) after 24 h of treatment. Nuclei were counterstained with Hoechst 33342 (blue). Representative images showing the OS cell line Saos-2 are shown. Nuclei of apoptotic cell appear light blue. **D** Quantification of the GSH/GSSG ratio in tumor- and control cell lines (*n* = 4 each) following treatment with 45S5-BG (0.5 mg/ml) for the indicated time. **E** ROS production in tumor and control cells in response to 45S5-BG (0.5 mg/ml). Treatment with 50 µM of the known ROS inducer Menadione (MD) for 6 h served as positive control. **F** LDH release in response to 45S5-BG treatment of tumor- and control cells (*n* = 4 each). (*p < 0.05 and **p < 0.01 compared to untreated cells).

In order to investigate the differential effects of BG particles on tumor- and non-neoplastic cells we compared two osteosarcoma (OS) and two chondrosarcoma (CS) cell lines with two primary human osteoblast-like (hOB) cell lines and two chondrocyte (CHO) cell lines, which served as non-neoplastic controls. If not otherwise stated, sintered 45S5-BG particles ( < 25 µm) at a concentration of 0.5 mg/ml were used for the experiments. As expected, the viability of all tested tumor cell lines significantly decreased to values below 15% within 48 hours, while the viability of CHOs and hOBs did not change significantly (Fig. 1A). Notably, the glass particles themselves, without cells, did not interfere with the WST-1 assay (Supplemental Fig. 1). Western blot analysis of cleaved caspase-3, the active form of caspase-3, PARP p85, the cleavage product of the caspase substrate PARP as well as the analysis of the caspase substrate NucView 488 by fluorescence microscopy confirmed the absence of caspase-3 or -7 activation in 45S5-BG treated cells indicating that apoptotic mechanisms are not involved in 45S5-BG mediated cell death (Fig. 1B–C). The levels of reduced glutathione (GSH) and oxidized glutathione (GSSG) were analyzed to determine the extent of BG-induced oxidative stress, with the GSH/GSSG ratio being indicative of the redox state of the cells. We observed a significant decrease of the GSH/GSSG ratio following 45S5-BG treatment indicating elevated oxidative stress that was, however, visible in both, tumor- and control cells (Fig. 1D). We further observed a rapid and significant increase in ROS levels in all tested cell types. which further confirms the BG-induced triggering of oxidative stress. (Fig. 1E). In order to detect any potential damage or loss of integrity of the plasma membrane caused by the 45S5-BG particles, we investigated the release of lactate dehydrogenase (LDH). Interestingly, a rapid increase of the LDH levels in the culture medium was induced by 45S5-BG exclusively in the tumor cells, suggesting a necrotic, ferroptotic or necroptotic cell death mechanism (Fig. 1F).

### 45S5-BG particles induce activation of the protein kinase AKT exclusively in tumor cells

**Fig. 2:**
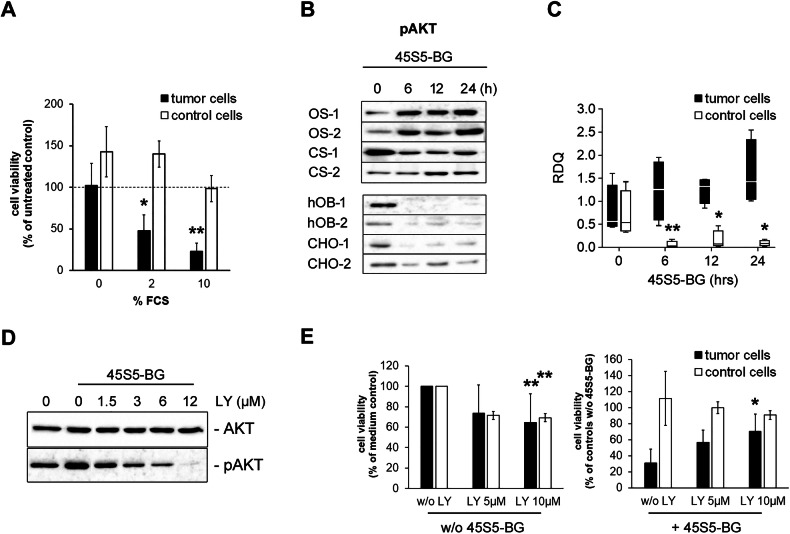
45S5-BG induced cell death requires activation of AKT kinase. **A** Viability of tumor- and control cells (*n* = 4 each) treated with 45S5-BG (0,5 mg/ml) for 24 h in culture medium containing 0, 2 and 10% FCS, respectively. Cell viability was measured by WST-1 assay and values are expressed as % of untreated cells (dashed line) (*p < 0.05 and **p < 0.01 compared to untreated cells). **B** Western blot analysis of AKT phosphorylation in response to 45S5-BG (0.5 mg/ml) in OS, CS, hOB and CHO cells. **C** Relative densitometric quantification (RDQ) of the western blots shown in **B** (*p < 0.05 and **p < 0.01 compared to untreated cells). **D** Western blot analysis of AKT and pAKT in osteosarcoma cells (Saos) treated with 45S5-BG (0.5 mg/ml) and increasing concentrations of the PI3K inhibitor LY294002 (LY) for 24 h. **E** Viability of tumor cells (*n* = 4) and control cells (*n* = 4) with and without addition of 45S5-BG (0.5 mg/ml) in combination with the PI3K inhibitor LY294002 (LY). (*p < 0.05 and **p < 0.01 compared to cells w/o LY).

During the analyses of BG-induced cytotoxicity, we noticed that the fastest-proliferating OS cells were the most sensitive. To confirm a possible correlation between growth rate and sensitivity towards 45S5-BG, we modulated the proliferation speed by using varying amounts of fetal calf serum (FCS) in the culture medium. Indeed, the cytotoxic effect of the BG particles was found to increase in a substantial manner with the FCS content of the medium. While tumor cells cultured in FCS-free medium were completely resistant to 45S5-BG treatment, the cell viability decreased significantly with increasing FCS concentrations (Fig. 2A). The serine/threonine kinase AKT, also known as Protein Kinase B or PKB, represents a central regulator of proliferation, cell cycle progression and survival. Western blot analyses of phosphorylated AKT revealed a moderate level of active AKT in all cell types. However, while AKT activity was significantly downregulated in hOB and CHO cells in response to 45S5-BG treatment it remained constant or was even upregulated in the analyzed tumor cell lines. (Fig. 2B–C). Accordingly, pretreatment of the cells with LY294002, a phosphatidylinositol 3-kinase (PI3K) inhibitor that impedes AKT activation, significantly inhibited AKT phosphorylation and the BG-induced cell death, while only slightly reducing cell viability itself (Fig. 2D–E).

### The capacity of bone tumor cells to undergo necroptosis is impeded by a deficiency in RIPK3

**Fig. 3:**
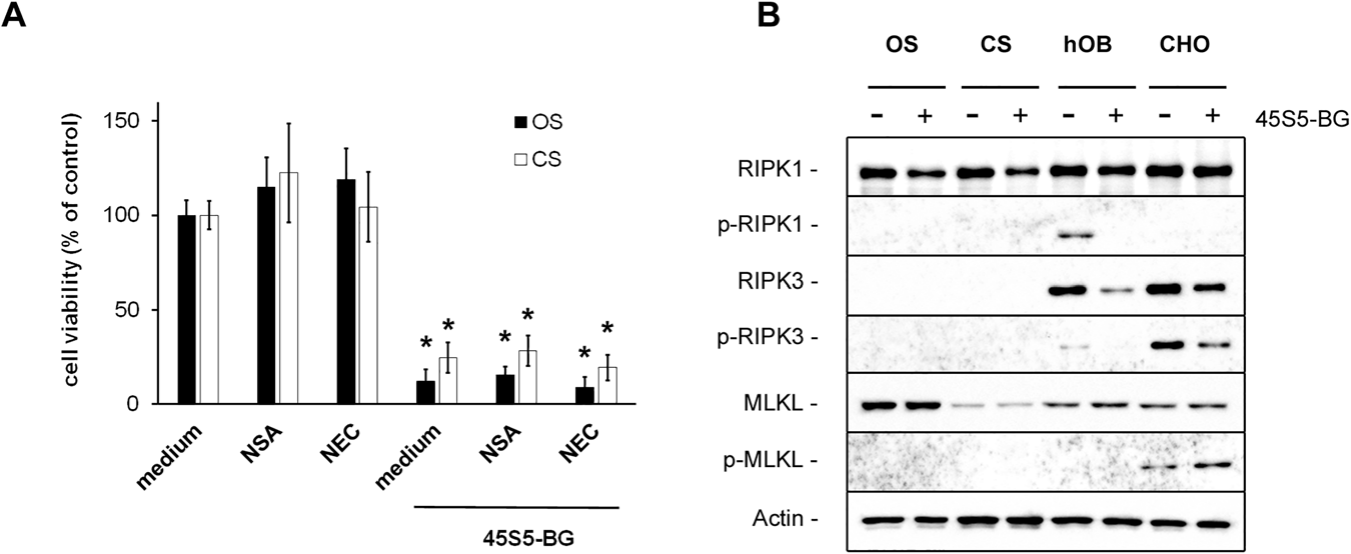
RIPK3 deficiency prevents the induction of necroptosis in bone tumor cells. **A** Cell viability of OS and CS cells after incubation with 45S5-BG particles ( < 25 µm, 0.5 mg/ml) with or without addition of the necroptosis inhibitors necrosulfonamid (NSA) (1 µM) or necrostatin-1 (NEC) (50 µM) for 48 h (*p < 0.05 compared to cells w/o 45S5-BG). **B** Western blot analysis of RIPK1, RIPK3, MLKL and their phosphorylated forms after treatment with 45S5-BG particles (0.5 mg/ml) for 24 h. The expression of Actin served as loading control.

Since the BG-induced cell death turned out to be caspase-independent and accompanied by a rupture of the plasma membrane, we investigated the possible involvement of necroptotic mechanisms. Both, Necrostatin-1 (NEC), a potent inhibitor of Receptor-Interacting protein kinases 1 (RIPK1) and Necrosulfonamide (NSA), a highly specific inhibitor of Mixed Lineage Kinase Domain-Like (MLKL) were ineffective in preventing 45S5-BG induced cell death (Fig. 3A). Western blot analysis of the key proteins that mediate necroptosis revealed that in contrast to the control cells, both, OS and CS cells do not express RIPK3 and are thus unable to phosphorylate MLKL, the decisive step in the necroptosis cascade. (Fig. 3B).

### Treatment with 45S5-BG particles selectively activates Mitogen-Activated Protein Kinases (MAPKs) in tumor cells

**Fig. 4:**
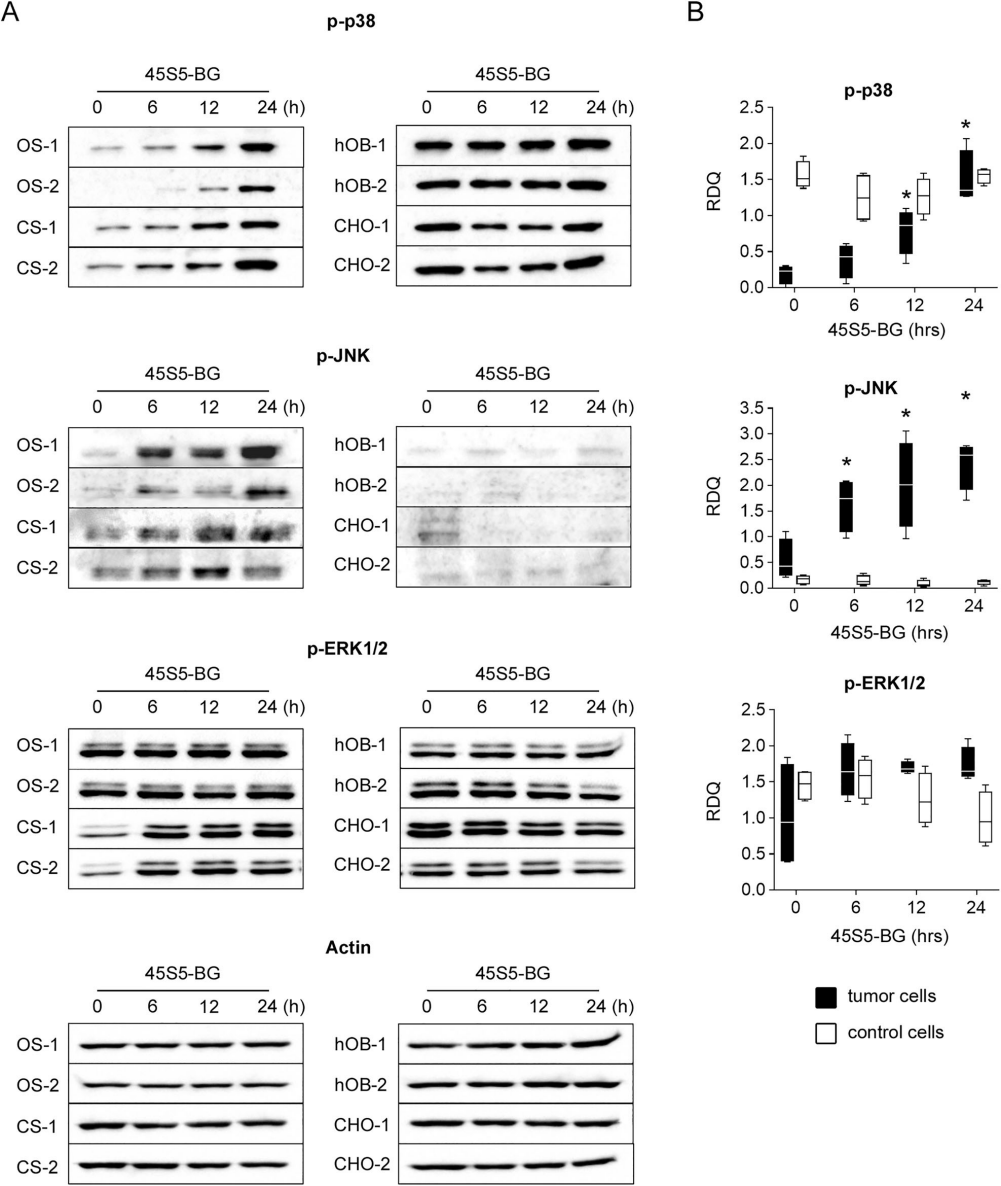
45S5-BG particles selectively induce phosphorylation of JNK and p38 in bone tumor cells. **A** Western blot analysis of phosphorylated p38, JNK and ERK1/2 in tumor- and control cells. Cells were treated with 45S5-BG particles ( < 25 µm, 0.5 mg/ml) for the indicated time before total proteins were isolated and subjected to Western blot analysis. Actin levels were used as loading controls and for normalization of the signals. **B** Relative densitometric quantification (RDQ) of signal intensities shown in **A**. Signals were normalized to the expression of Actin in the corresponding sample. The mean values of tumor- and control cells are indicated by lines, the upper and lower boundaries of the boxes indicate the 75^th^ and 25^th^ percentile. The whiskers indicate the highest and lowest values (*p < 0.05 compared to untreated cells).

Since MAPKs play important roles in the transmission of extracellular stimuli, we investigated their activation in response to BG treatment. Western blot analysis of the phosphorylated (activated) MAPKs p38, extracellular signal-regulated kinase (ERK1/2) and c-Jun N-terminal kinase (JNK) were performed after treatment of tumor- and control cells with 45S5-BG particles for 6, 12 and 24 h. While the control cells exhibited consistent p38 activity, that was not influenced by the BG particles, tumor cells displayed initially low p38 activity that increased up to eightfold within 24 h of BG treatment. While JNK activity was nearly undetectable in the control cell lines, treatment with 45S5-BG resulted in a rapid and substantial increase in JNK activity in tumor cells, reaching 4.4-fold higher levels within 24 h. Conversely, ERK1/2 activity was consistently observed in all cell types analyzed, exhibiting constant levels in control cells and only a marginal increase in tumor cells (Fig. 4A–B).

### Bone tumor cells, but not control cells, release ATP in response to 45S5-BG in a MAPK dependent manner

**Fig. 5:**
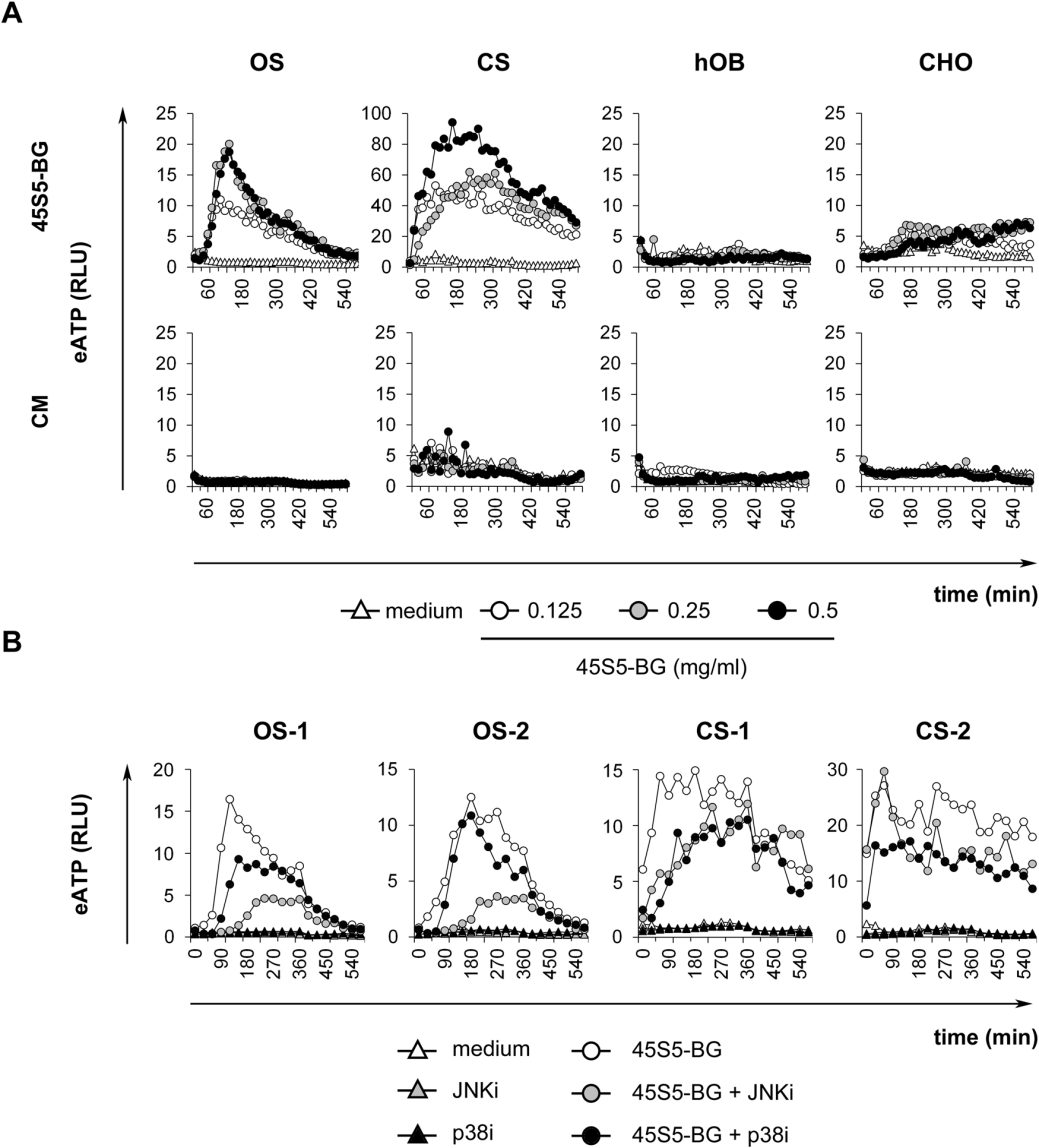
45S5-BG induces a rapid and MAPK-dependent release of ATP from tumor cells. **A** Kinetics of ATP release from OS, CS, hOB and CHO cells in response to 45S5-BG particles ( < 25 µm) and conditioned medium (CM), respectively. Levels of eATP in the supernatants are presented as relative luminescence units (RLU). **B** Quantification of eATP in response to 45S5-BG (0.5 mg/ml) with or without co-treatment with the JNK inhibitor SP600125 (25 µM) and the p38 inhibitor SB202190 (25 µM), respectively.

Following the observation of LDH release in response to 45S5-BG treatment, we aimed to identify the release of further factors that might function as Damage-Associated Molecular Pattern (DAMPs). As ATP has already been shown to function as a DAMP, the concentration of extracellular ATP (eATP) in the culture medium was determined in 30-minute intervals for 10 h. The cells were treated with increasing amounts of 45S5-BG particles ( < 25 µm) or alternatively with culture medium that had been conditioned with an equivalent quantity of 45S5-BG for a period of 24 h before BG particles were removed by centrifugation. This approach was adopted in order to analyze the influence of the ions released by the BG. We observed a rapid and significant release of ATP from tumor cells, which peaked at approximately 3 h following 45S5-BG treatment and was absent in control cells. The conditioned medium was found to be incapable of inducing this release, thereby indicating that the ions released from BGs are not involved in this process (Fig. 5A). Interestingly, both, inhibition of JNK and p38, suppressed the 45S5-BG-induced ATP release, with the JNK inhibitor demonstrating the most significant effect, particularly in osteosarcoma cells. These data prove that the BG-induced ATP release is a MAPK-dependent process (Fig. 5B).

### Treatment with 45S5-BG induces an increase in intracellular calcium concentrations in tumor cells

**Fig. 6:**
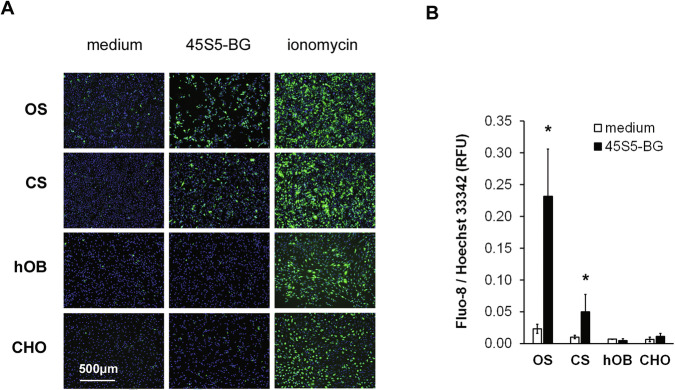
45S5-BG induces an increase of intracellular calcium in tumor cells but not in control cells. **A** Representative images of OS, CS, hOB and CHO cells stained with Fluo-8 (green) after treatment with 45S5-BG ( < 25 µm, 0.5 mg/ml) for 24 h. The calcium ionophore ionomycin (2 µM) served as positive control. Cells were counterstained with Hoechst 33342 (1 µg/ml) (blue). **B** Relative quantification of calcium levels using ImageJ. The Hoechst 33342 fluorescence was used for normalization (*p < 0.05 compared to untreated cells).

Whether 45S5-BG particles have the capacity to induce a modulation of intracellular calcium levels was investigated using the fluorescent calcium indicator Fluo-8. A semi-quantitative analysis of fluorescence microscopy images revealed a strong increase of calcium in OS cells, representing the most sensitive cells towards 45S5-BG treatment, and a moderate increase in CS cells. In contrast, the control cells showed no alteration in intracellular calcium concentrations in response to 45S5-BG (Fig. 6A–B).

### The 45S5-BG particles form a close association with the cells

**Fig. 7:**
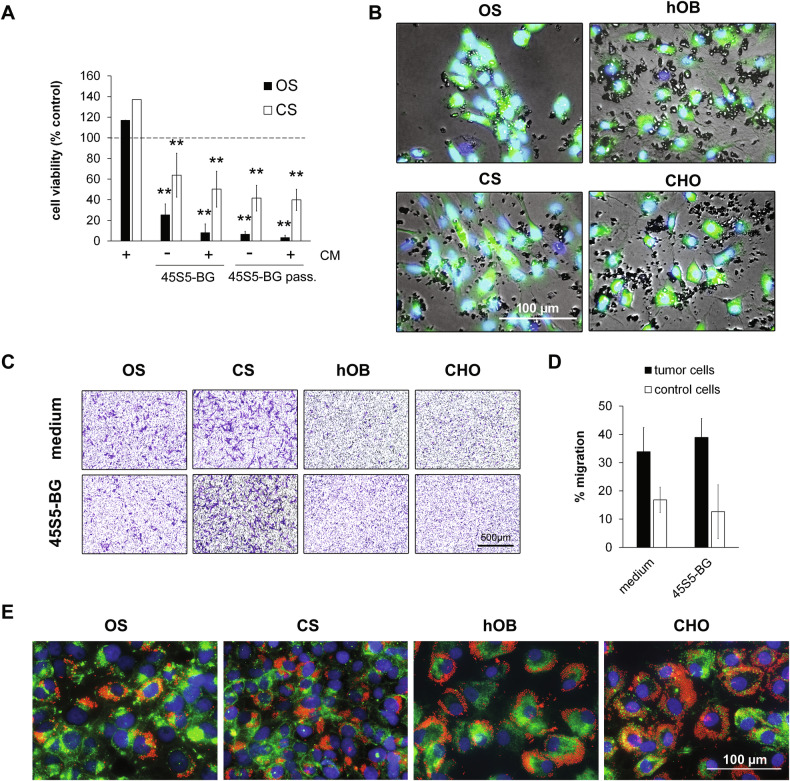
Physical interactions of 45S5-BG and silica particles with tumor- and control cells. **A** Quantification of tumor cell viability after treatment with 45S5-BG-conditioned medium (CM) with or without the addition of unmodified 45S5-BG particles or passivated 45S5-BG particles (*p < 0.05 and **p < 0.01 compared to untreated cells – dashed line). **B** Representative brightfield images of OS, CS, hOB and CHO cells incubated with 45S5-BG particles for 12 h. Cells were washed to remove unbound BG-particles and stained with calcein-AM (green) and Hoechst 33342 (blue). Merged images are shown. **C** Representative images of cells stained with crystal violet after migration through trans-well inserts with a pore size of 8 µm. Cells were attracted with culture medium supplemented with 1% FCS with or without 45S5-BG particles (0.5 mg/ml). **D** Densitometric quantification of migrated cells using ImageJ software. **E** Representative fluorescence images of tumor- and control cells after incubation with PE-labeled silica particles (red) for 12 h. Cell membranes were visualized using CellBrite Green (green) and nuclei were counter-stained with Hoechst 33342 (blue).

To investigate the significance of a direct contact between BG particles and cells, and to further clarify the impact of the ions released from the BG particles for the induction of cell death, tumor cells were treated with conditioned media (CM). The CM was prepared by incubating 45S5-BG particles in cell culture medium for 24 h, after which the particles were removed by centrifugation. In addition, we investigated the capacity of the passivated particles produced during CM preparation to trigger cell death. We observed that the CM was incapable of either inducing cell death by itself or enhancing the level of cell death induced by 45S5-BG. Further, passivation of the BG did not result in a reduction of the cytotoxic effect (Fig. 7A). These data illustrate the importance of direct interaction between the particles and the cells. In fact, we observe a rapid attachment of particles to the cells, independent from the type of cell (Fig. 7B). However, 45S5-BG was not able to accelerate the migration speed of the cells in a trans-well assay, excluding an active movement of the cells towards the BG particles (Fig. 7C–D). Whilst the majority of the particles appeared to adhere to the surface of the cells, the possibility of the smaller particles entering the cells was the subject of our further investigations. In order to enhance the efficacy of the tracking process, we used phycoerythrin (PE)-labeled silica particles, characterized by a uniform size of 1.5 µm and spherical shape. To facilitate the localization of the particles, the cell membranes were stained with the fluorescent dye CellBrite Green. All tested cell lines, tumor- and control cells, showed a massive uptake of the particles. After 12 h of incubation the majority of particles was located in the cytoplasm surrounding the nuclei (Fig. 7E).

### 45S5-BG particles induce ferroptosis in bone tumor cells

**Fig. 8:**
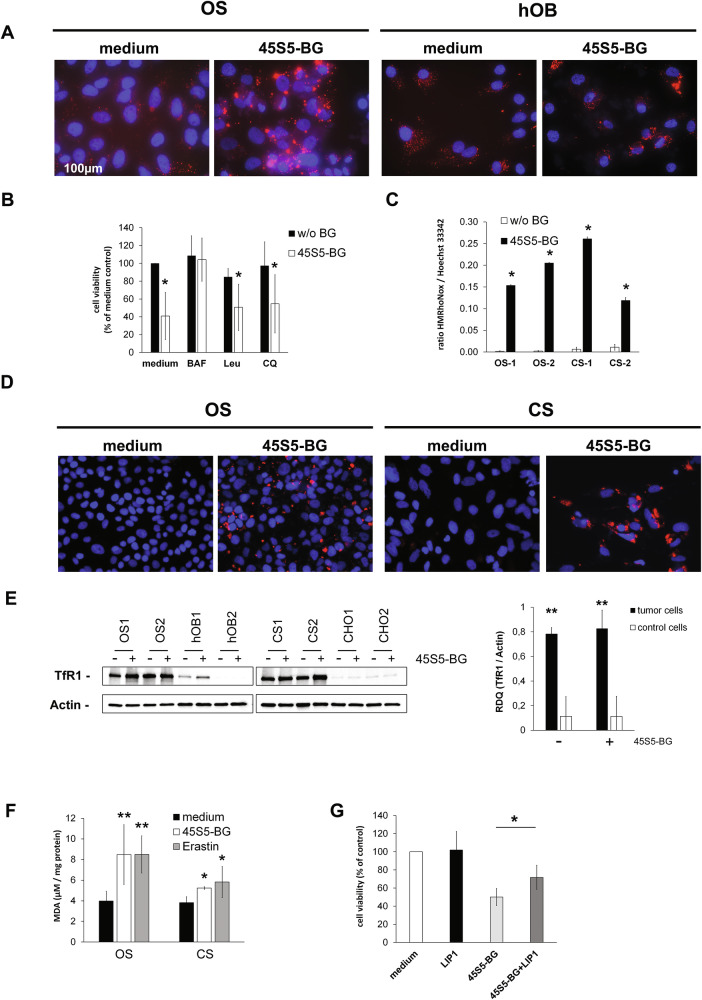
45S5-BG particles induce ferroptosis in bone tumor cells. **A** Fluorescent staining of lysosomes in tumor- and control cells with or without 45S5-BG treatment for 24 h. Cells were stained with LumiTracker LysoRed (red) and counterstained with Hoechst 33342 (blue). Representative images of tumor- (OS) and control cells (hOB) are shown. **B** Analysis of cell viability of tumor cells (OS and CS) after treatment with 45S5-BG (0.5 mg/ml) for 24 h with or without the addition of 50 nM Bafilomycin A1 (BAF), 50 µM Leupeptine (Leu) and 25 µM Chloroquine (CQ) (* p < 0.05 compared to untreated cells). **C** Quantification of redox-active Fe^2+^ in the labile iron pool (LIP) in OS and CS cell lines. Cells were stained with HMRhoNox (red) and counterstained with Hoechst 33342 (blue) for normalization. Signal intensities were quantified using ImageJ software (*p < 0.05 compared to untreated cells). **D** Representative images of HMRhoNox-stained OS and CS cells with and without 45S5-BG treatment. **E** Western blot analysis of the TfR1 expression in untreated and 45S5-BG treated tumor- and control cells and semiquantitative evaluation of the TfR1 signal intensities. Data were normalized to the expression of Actin in the corresponding sample (*p < 0.05 and **p < 0.01 compared to control cells). **F** Quantification of Malondialdehyde (MDA) in OS and CS cells after treatment with 45S5-BG (0.5 mg/ml) or Erastin (1 µM) for 24 h. Data were normalized to the protein levels (*p < 0.05 and **p < 0.01 compared to untreated cells). **G** Cell viability of tumor cells (OS and CS) after treatment with 45S5-BG (0.5 mg/ml) for 24 h with or without the addition 10 µM Liproxstatin-1 (LIP1) (* p < 0.05).

The uptake of particles into the cells indicated the involvement of lysosomes. In fact, we observed an increase in the number and size of lysosomes in response to 45S5-BG in both analyzed cell types that was, however, more pronounced in tumor cells (Fig. 8A). In order to further investigate the importance of the lysosomes for the BG-induced cell death, the impact of several lysosome-related compounds was analyzed. Bafilomycin A1 is a highly specific inhibitor of the ATP-dependent V-ATPase, which is responsible for the transportation of protons into lysosomes, thereby reducing the pH-value within these organelles. Chloroquine has been shown to enter the lysosomes by diffusion where it is trapped by protonation and functions as weak base and thus also elevates the pH value. Leupeptin, on the other hand, functions as a lysosomal inhibitor by blocking key proteases within the lysosomes, including cathepsins B and L, thereby slowing down or preventing the degradation of proteins and organelles, which is crucial for processes like autophagy and intracellular digestion. Among these substances only Bafilomycin A1 was capable of impeding the 45S5-BG-induced cellular toxicity (Fig. 8B).

The necessity of an intact V-ATPase activity that is also known to be crucial for maintaining iron homeostasis pointed to a possible involvement of ferroptosis. We therefore analyzed the amount of Fe²⁺ in bone tumor cells in response to 45S5-BG. A semi-quantitative analysis using the Fe²⁺-selective fluorescent probe HMRhoNox-M showed that the fluorescence intensity increased by up to 100-fold after 24 h of treatment, indicating a significant increase in iron concentration. (Fig. 8C–D). Western blot analysis of transferrin receptor 1 (TfR1), a transmembrane glycoprotein primarily responsible for cellular iron uptake, revealed that 45S5-BG treatment did not influence receptor expression. However, the constitutive expression of this receptor was found to be 6.8-fold higher in untreated tumor cells compared to the control cells. (Fig. 8E). We then analyzed the accumulation of Malondialdehyde (MDA), a stable end-product and reliable biomarker of lipid peroxidation, the key event during ferroptosis. Treatment of OS and CS cells with 45S5-BG resulted in significantly increased MDA levels, comparable to those observed following treatment with Erastin, a well-known ferroptosis inducer (Fig. 8F). To further confirm BG-induced ferroptosis, tumor cells were treated with 45S5-BG, either alone or in combination with liproxstatin-1 (LIP1). LIP1 is an antioxidant which inhibits ferroptosis by reducing lipid peroxidation. Treatment with LIP1 resulted in a significant reduction in BG-induced cell death after 24 h (Fig. 8G). Together, these data strongly suggest the induction of ferroptosis by 45S5-BG with an elevated TfR1 expression as possible cause for the observed higher sensitivity of tumor cells.

## Discussion

Despite advances in treatment modalities, current bone tumor therapies still face significant challenges, including severe side effects of conventional approaches, poor survival rates and high rates of tumor recurrence. We previously identified a strong cytotoxic effect of 45S5-BG particles on bone tumor cells, which was absent in non-malignant mesenchymal stromal cells [13]. We further observed that the size of the particles and sintering of the material are significant factors in determining the extent of the cytotoxic effect, with sintered particles measuring less than 25 µm proving to be the most effective [14]. We could also demonstrate a very rapid and sustained burst release of silicon, calcium, and phosphorus ions into the culture medium; that was, however, not necessary for the cytotoxic effect [15].

In the present study, we aimed to identify the mechanisms that mediate the observed tumor cell-specific cytotoxicity using OS and CS cell lines in comparison to primary osteoblast-like cells and chondrocytes, their non-neoplastic counterparts. Independent from the cell type, BG treatment induced oxidative stress as indicated by increased ROS levels and decreased GSH/GSSG ratios. Although GSH depletion is a consistent and early hallmark of apoptosis [16], we could exclude apoptosis as possible cause of BG-induced cell death. However, the failure to maintain the bioenergetic status, often stemming from glutathione imbalance, can also result in the morphological shift toward necrosis or regulated necroptosis [17]. In addition, a critical depletion of the total glutathione pool below a necessary functional threshold, may result in the failure of the antioxidant defense systems leading to the lethal accumulation of lipid ROS in an iron-dependent manner known as ferroptosis [18].

As BG-induced cell death was found to be caspase-independent and associated with cell membrane rupture and subsequent LDH release, we investigated whether necroptosis could be the mechanism behind the observed 45S5-BG-induced cell death. The core mechanism of necroptosis is mediated by the Receptor-Interacting protein kinases 1 and 3 (RIPK1 and RIPK3) and the Mixed Lineage Kinase Domain-Like (MLKL) [19]. Several studies have already demonstrated that micro- and nano-plastics can upregulate the expression of RIPK1, RIPK3 and MLKL, leading to necroptosis [20]. However, the inability of RIPK1 and MLKL inhibitors to prevent 45S5-BG-induced cell death, suggests that this process is not associated with necroptosis. The absence of RIPK3 expression observed in bone tumor cells, which is probably due to the epigenetic silencing of RIPK3 previously observed in human osteosarcoma [21], further contradicts the involvement of necroptosis.

Amongst the tumor cell-specific effects induced by BG-treatment, we further identified the activation of MAPKs. MAPKs are involved in many signal-transduction cascades within the cell and their main function is to transmit signals from the cell surface to the nucleus to trigger specific cellular responses, including cell death [22]. They are activated by various extracellular stimuli including growth factors and cytokines but also cellular stressors such as osmotic shock and UV radiation. Interestingly, nanoparticles have also been shown to induce oxidative stress and toxicity [23–25]. In our study, we demonstrated that the activation of JNK and p38 is necessary for BG-induced cell death, and that these kinases are essential for the rapid release of ATP from tumor cells triggered by BG particles. Extracellular ATP may reflect both, the consequence of the 45S5-BG induced cell death as well as a potential cause of cell death. It is recognized as a Damage-Associated Molecular Pattern (DAMP) and has been shown to be actively secreted into the extracellular space by dying tumor cells where it acts as danger signal for the immune system [26]. On the other hand, high concentrations of extracellular ATP can also directly induce cell death. The binding of high concentrations of eATP to ATP-gated ion channels, primarily the P2X7 receptor, can lead to an overstimulation. This overstimulation has been shown to induce the formation of a large, non-selective pores in the cell membrane that disrupt ion homeostasis, especially by allowing the massive influx of calcium ions which finally triggers cell death [27, 28]. The observed increase of calcium levels observed in response to BG treatment may thus be triggered by elevated extracellular ATP. ATP is known to bind to P2X (ligand-gated ion channels) and P2Y (G-protein coupled) receptors on the cell surface. Activation of these receptors has been shown to cause an influx of calcium ions from the extracellular space, as well as the release of sequestered calcium from internal stores, such as the endoplasmic reticulum and lysosomes [29, 30]. A rapid calcium influx can then lead to cellular swelling, membrane rupture, uncontrolled cell death and has also been implicated in the regulation of autophagic cell death [31, 32]. Intracellular calcium overload has further been shown to trigger ROS production, which directly promotes lipid peroxidation [33]. Thus, calcium acts as a critical mediator in the induction and execution of ferroptosis, particularly by acting as a bridge between iron overload, mitochondrial dysfunction, and lipid peroxidation [34]. However, our data do not show whether the observed BG-induced increase in intracellular calcium concentration is directly related to the induction of ferroptosis.

It is important to note that we previously demonstrated the necessity of a direct contact between BG-particles and the cells for the induction of cell death. Neither the treatment of tumor cells with BG-conditioned medium nor the separate cultivation of cells and BG particles in a trans-well system was effective in inducing cell death [15]. Accordingly, we also observed a close association of cells and BG particles in this study. A possible explanation for the need of a direct contact between the particles and the cells may be the assumption that local ion concentrations at the particle-cell interface significantly exceed those in the medium. This could lead to the attainment of a critical threshold necessary for the cytotoxic effect. However, we observed in previous studies that a change in glass composition—and thus a change in the ion release profile—has no significant effect on cytotoxicity. Furthermore, in the current study, we demonstrate that passivated glass, with significantly reduced ion release, still exhibits full cytotoxicity. Thus, ion release seems to plays only a minor, if any, role in cytotoxicity. In addition, at least for SiO_2_-particles, we were able to prove our assumption that the particles not only adhere to the cell surface but are also absorbed by the cells. Although these observations are not fully transferable to the 45S5-BG particles, which has to be considered as limitation of our study, cellular uptake of nano- and micro-sized particles has already been shown for different materials and cell types. In accordance with our results, FITC-conjugated silica particles have been shown to penetrate primary fibroblasts and HeLa cells in large numbers [35]. Mesoporous silica particles have been shown to enter the cells via endocytosis and consequently localize within lysosomes [36, 37]. The need for internalization of the BG particles could also explain our previous observations that smaller particles exhibit greater cytotoxicity and that ion release does not play a decisive role.

Colocalization with lysosomes has also been shown for modified mesoporous BG particles that were able to release their ionic components intracellularly, suggesting a possible role for the delivery of therapeutic substances [38]. Lysosomes are filled with acid hydrolases and function as intracellular recycling centers. The accumulation of particles within the lysosomes is a critical event that can lead to their damage in form of a lysosomal membrane permeabilization (LMP). LMP is characterized by the release of the lysosomal content including calcium, iron and hydrolases into the cytosol, resulting in osmotic lysis or direct membranolytic activity of the compounds [39, 40]. The observed increase in lysosome number in response to BG treatment may reflect the adaptive response of the cells to the heavy particle load, while the increase in size suggests a fusion of the lysosomes and the formation of endolysosomes or autophagosomes. In terms of cell specificity, it is important to note that the lysosomes of tumor cells exhibit lower membrane stability compared to non-neoplastic cells [41]. This predisposed vulnerability makes tumor cells particularly susceptible to particle-induced LMP, whereas the more robust lysosomes of healthy cells can withstand the same stress. Lysosomal stability is not constant, but subject to changes caused by degradation and repair. Lysosomal dysfunction has also been shown to be an age-related process [42, 43]. Therefore, variability among the donors from whom the primary control cells were isolated may affect lysosomal stability. Primary control cells were therefore isolated from patients of a comparable age and used in a passage-matched manner. The fact, that leupeptin was unable to inhibit 45S5-BG-induced cell death in our study indicates that the release of proteolytic enzymes from lysosomes is not the causative factor for the observed cell death, although it is a common event during LMP [44]. The failure of chloroquine to inhibit cell death indicates that an alkalization of lysosomes alone is inadequate to induce cell death. On the other hand, the inhibitory effect of Bafilomycin A1 demonstrates that an intact V-ATPase activity is essential for the BG-induced cell death. Interestingly, V-ATPase activity is known to be crucial for maintaining iron homeostasis and inhibition by Bafilomycin A1 has been shown to inhibit the iron-dependent ferroptosis [45]. Ferroptosis is a regulated, non-apoptotic cell death mechanism driven by iron accumulation, lethal lipid oxidation and subsequent membrane rupture. Since ferroptosis is further characterized by oxidative stress, a reduction of GSH levels and increased ROS levels, our data strongly suggest a V-ATPase driven lysosomal ferroptotic cell death triggered by 45S5-BG particles. The main source of the redox-active iron that drives ferroptosis is the ferrous iron (Fe^2+^) found in the intracellular labile iron pool (LIP), which is located in the cytosol, mitochondria and lysosomes and is replenished by the uptake of extracellular iron via transferrin receptors (TfR1). Due to their high proliferation rate and metabolic activity tumor cells have a high demand for iron that is often achieved by overexpression of TfR1. Thus, TfR1 is a positive regulator of ferroptosis and can sensitize cells to ferroptosis by the increased iron uptake [46]. Accordingly, we observed significantly higher constitutive TfR1 expression in tumor cells and a massive increase of intracellular Fe^2+^ in response to 45S5-BG treatment. To finally confirm ferroptosis as the underlying mechanism of 45S5-BG induced cell death, we verified a significant accumulation of Malondialdehyde (MDA), a stable end-product and reliable biomarker of lipid peroxidation, the key event during ferroptosis that finally leads to membrane rupture and cell death [47].

An important aspect of tumor cell selectivity for BG particles may be related to their fundamentally altered metabolic properties compared to healthy cells. Tumor cells exhibit disrupted metabolic activity geared toward high proliferation and growth potential [48]. This altered metabolism, often referred to as the Warburg effect, leads to elevated levels of endogenous oxidative stress. Mitochondria, responsible for energy production, are often dysregulated in tumor cells, further increasing their susceptibility to oxidative stress [48]. The BG particles might exploit this molecular vulnerability. While healthy cells possess robust antioxidant defense systems and are less affected by particle-induced stress, the already stressed tumor cells quickly enter a critical state that shifts the balance toward irreversible, fatal cell death. Our observations of reduced BG cytotoxicity after FCS withdrawal or AKT inhibition, and thereby reduced cell proliferation, supports this hypothesis.

Knowledge of the mechanisms that mediate BG-induced cell death, coupled with the ability to modulate BG properties by altering their composition, enables the development of new therapeutic strategies targeting both, bone repair and tumor relapse. The therapeutic potential of BG-particles is further increased by our finding that all analyzed tumor cell lines exhibited a highly comparable response to BG treatment, indicating that cell death induction is independent of the type of tumor.

## Materials and methods

### Cell lines, isolation and culture

The human osteosarcoma (OS) cell lines HOS 143B (Sigma-Aldrich, Taufkirchen, Germany) and Saos-2 (Cell Line Service GmbH, Eppelheim, Germany) were cultured in Dulbecco’s Modified Eagle’s Medium (DMEM) high glucose (Thermo Fisher Scientific, Karlsruhe, Germany) supplemented with 5% fetal calf serum (FCS) (Bio&Sell GmbH, Nürnberg, Germany) and 100 U/ml penicillin/streptomycin (Sigma-Aldrich). The chondrosarcoma (CS) cell lines SW1353 (Cell Line Service GmbH), and CAL-78 (DSMZ, Braunschweig, Germany) were cultured in DMEM / F12 medium supplemented with 10% FCS (Bio&Sell) and 100 U/ml penicillin/streptomycin (Sigma-Aldrich). Culture flasks were coated with 37.5 µg/ml collagen Type I (Sigma-Aldrich) diluted in 0.1% acetic acid for 30 min prior to cell seeding. Authentication of these cell lines was performed by STR analysis by the companies. As non-neoplastic control cells served two primary human osteoblast-like cell lines (hOB) and two human chondrocyte cell lines (CHO) that were isolated from patients with diagnosed osteoarthritis of the knee and underwent a complete knee replacement surgery as described previously [14]. The age of the patients ranged from 61–67 years, and the cells were used passage-matched (passages 2–3) for the experiments. CHO cells were cultured in CS medium and primary hOBs were cultured in DMEM low glucose (Thermo Fisher Scientific) containing 1 g/l glucose, 10% FCS (Bio&Sell GmbH), 100 U/ml penicillin/streptomycin (Sigma-Aldrich), and 0.17 mM ascorbic acid 2-phosphate (Sigma-Aldrich). The cell lines were regularly tested for mycoplasma contamination.

### BG production

The bioactive glass 45S5-BG with the chemical composition SiO_2_ (46.1%), CaO (26.9%), Na_2_O (24.4%), and P_2_O_5_ (2.6%) (all in mol%) was produced by the melt-quenching method as detailed elsewhere [15]. The glass was ground with a planetary ball mill (Retsch, Germany) and the desired fraction with a particle size <25 µm was obtained by sieving. Sintering was carried out by heat treatment at 1050 °C for 2 h. The particle size distribution within this fraction was obtained by laser diffraction using the Mastersizer 3000 (Malvern Panalytical, UK), resulting in the following measurements: D10 = 5.6 ± 0.2 µm, D50 = 17.0 ± 0.2 µm and D90 = 36.2 ± 2.4 µm. The characterization of the glass particles was described in our previous study [14]. For the analysis of particle internalization, fluorescently labeled silica particles with a diameter of 1.5 µm were used (Kisker Biotech, Steinfurt, Germany).

### Cell viability assay

The assessment of cell viability was conducted by means of a WST-1 assay (Santa Cruz Biotechnology, Heidelberg, Germany) as described previously [14]. In brief, 10,000 cells per well were plated in 96-well cell culture plates and exposed to 45S5-BG (0.5 mg/ml) for the indicated time. When investigating the influence of specific substances, such as inhibitors, they were added 3 h prior to the addition of the BG particles. The reaction was quantified in a microplate reader (Autobio-Phomo, Anthos Microsystems, Freising, Germany) at 450 nm with a reference wavelength of 600 nm. Wells without cells were used as blanks and subsequently subtracted from the experimental samples. All measurements were conducted at least in duplicates.

### GSH/GSSG assay

Determination of the oxidative stress level of the cells was done by quantification of reduced glutathione (GSH) and oxidized glutathione (GSSG). Analysis was done using the GSH/GSSG-Glo kit from Promega (Promega GmbH, Walldorf, Germany) according to the manufacturers protocol. In brief, 10,000 cells per well were plated in 96-well cell culture plates and exposed to 45S5-BG (0.5 mg/ml) for 6, 12 and 24 h before cells were lysed. After the addition of a luciferine generation reagent and a luciferine detection reagent the luminescence was measured in a FluoStar microplate reader (BMG LabTech, Ortenberg, Germany). The GSH and GSSG concentrations were calculated from standard curves and the GSH/GSSG ratios were calculated as a measure for oxidative stress.

### ROS detection

Reactive oxygen species (ROS) were detected using the ROS-Glo H₂O₂ Assay from Promega, following the manufacturer’s instructions. The cells were seeded into 96-well cell culture plates at a density of 15,000 cells per well. Adherent cells were treated with 45S5-BG particles (0.5 mg/ml) and a specific H₂O₂ substrate that was added six hours before the end of the desired incubation period. After addition of 100 µl of a ROS-Glo detection solution and a final incubation at room temperature for 20 min, the relative luminescence was recorded using a FluoStar microplate luminometer (BMG LabTech). Wells without cells were used as blanks and subtracted from the test samples.

### LDH release assay

Plasma membrane damage was monitored by the release of lactate dehydrogenase (LDH) from the cells into the cell culture medium. The quantification of LDH was conducted using the LDH-Glo Cytotoxicity Assay from Promega, following the manufacturer’s instructions. In summary, 10,000 cells per well were plated in 96-well cell culture plates and treated with 45S5-BG (0.5 mg/ml) for 12, 24 and 48 h. The generated relative luminescent signal that is proportional to the quantity of LDH present in the culture supernatant was recorded using a FluoStar microplate reader (BMG LabTech). LDH levels were calculated using a standard curve.

### Detection of active Caspase 3/7

The activation of caspases was quantified using the caspase substrate NucViev 488 (Biomol, Hamburg, Germany), which stains the nuclei of apoptotic cells bright green upon cleavage by active caspase-3/7, a hallmark of apoptosis. Cells were seeded at a density of 20,000 cells per well in black 96-well cell culture plates with a clear bottom. On the next day, medium was replaced by fresh medium with or without 45S5-BG (0.5 mg/ml). Cells treated with the known apoptosis inducers Cisplatin (CPP) (5 µg/ml) and Staurosporine (STS) (200 nM) served as positive controls. Following treatment, cells were stained with NucView 488 (2.5 µM), diluted in Hanks’ balanced salt solution (HBSS) (Sigma-Aldrich) for 30 min at 37 °C and counterstained with 1 µg/ml Hoechst 33342 (Thermo Fisher Scientific) for 10 min at 37°C. The stained cells were photographed using a BZ-X800 microscope (Keyence, Neu-Isenburg, Germany).

### Western Blot analysis

Total proteins for western blot analysis were isolated from 500,000 cells cultured in 25 cm^2^ culture flasks. Following the desired treatment, cells were washed twice with phosphate buffered saline (PBS) and lyzed with 250 µl of RIPA buffer (Santa Cruz Biotechnology) supplemented with a proteinase/phosphatase inhibitor cocktail (Cell Signaling, Frankfurt, Germany). A quantity of 10 µg of total proteins was separated on a 10% polyacrylamide gel, transferred to an Immobilon-P membrane (Millipore, Schwalbach, Germany) and blocked with 5% non-fat dried milk (Sigma-Aldrich) diluted in TBS-T (TBS buffer supplemented with 0.1% Tween 20) (Sigma-Aldrich). The membranes were then subjected to an overnight incubation at 4 °C with the following primary antibodies diluted in TBS-T buffer containing 3% non-fat dried milk: Actin (1:5000) (BD Biosciences, Heidelberg, Germany), AKT, pAKT, RIPK1, pRIPK1, RIPK3, pRIPK3, MLKL, pMLKL, P38, pP38, JNK, pJNK, Erk1/2, pErk1/2 and CD71/TfR1 (all 1:1000; Cell Signaling). Following three washes in TBS-T, membranes were incubated for 1 h at room temperature with peroxidase conjugated secondary antibodies (1:5000; Dianova, Hamburg, Germany). Signals were detected using the chemoluminescence substrate Clarity (Bio-Rad Laboratories, München, Germany). Actin signals were used for normalization. Signal intensities were quantified using ImageJ software version 1.54 (http://imagej.net/ij/). Uncropped western blots are shown in Supplemental data / Original data.

### Quantification of extracellular ATP

Extracellular ATP (eATP) was quantified using a RealTime-Glo Extracellular ATP Assay (Promega). Cells were seeded in 96-well culture plates (10,000 cells/well) and incubated for 16 h at 37 °C before medium was replaced by fresh medium supplemented with 45S5-BG particles (0.5 mg/ml) or conditioned media (CM). Following the addition of a 4x RealTime-Glo™ Assay Reagent the eATP dependent luminescence was measured every 30 min in a FluoStar microplate reader (BMG LabTech) for a total period of 10 h. For the experiments in which the influence of MAPK inhibitors was analyzed, they were added one hour prior to the addition of 45S5-BG.

### Semi-quantitative analysis of intracellular calcium levels

Intracellular calcium mobilization was analyzed using the fluorescent probe Fluo-8 (Biomol). Fluo-8 is a cell-permeable dye whose fluorescence is greatly enhanced upon binding to calcium. Cells were plated in black 96-well plates with clear bottom (15,000 cells/well) and incubated for 16 h at 37 °C before 45S5-BG (0.5 mg/ml) or the calcium ionophore Ionomycin (2 µM) that served as positive control were added. Following 24 h of treatment, the medium was replaced by fresh culture medium supplemented with Fluo-8 (4 µM) and further incubated for 1 h at 37 °C. During the final 10 min of incubation the DNA dye Hoechst 33342 was added to a final concentration of 1 µM. Green (Fluo-8) and blue (Hoechst 33342) fluorescence was documented using a Keyence BZ-X810 microscope (Keyence) and quantified using ImageJ software version 1.54 (http://imagej.net/ij/). The Hoechst 33342 counterstain was used for normalization.

### Cell migration assay

Cell migration was analyzed using a trans-well system based on 24-well inserts with PET membranes with a pore size of 8 µm (SABEU GmbH, Northeim, Germany). The cells were subjected to a 24 h serum starvation period, followed by trypsinization, cell counting, and resuspension in cell culture medium containing 1% FCS. Subsequently, 25,000 cells were seeded into the upper chamber of the trans-wells, with a volume of 200 µL, while the lower chambers were filled with 600 µl cell culture medium (1% FCS) with or without the addition of 45S5-BG (0.5 mg/ml). The cells were incubated at 37 °C for 16 h, fixed and stained with crystal violet (0.125% in 50% methanol) and photographed (total cell number). Non-migrated cells in the upper chamber were wiped off the membrane with a cotton swab before the migrated cells on the inferior surface of the membrane were photographed. Three images were taken per well for total and migrated cells. The stained area was determined using ImageJ software, version 1.54, and the percentage of migration was calculated.

### Detection of intracellular Fe^2+^

Fe^2+^ levels were detected using the Fe^2+^-selective fluorescent probe HMRhoNox-M. Cells were seeded on 8-well chamber slides (Sarstedt, Nümbrecht, Germany) at 20,000 cells/well and incubated for 24 h until the cells adhered. Following the desired treatment, the cells were incubated for 30 min in serum-free cell culture medium, washed three times in HBSS buffer and stained with 1 µM HMRhoNox-M diluted in HBSS buffer for 30 min. The cells were then washed again three times for 5 min with HBSS and counterstained for 10 min with Hoechst 33342. Three images were taken from each well using a Keyence BZ-X810 microscope (Keyence). Fluorescence intensities were analyzed using ImageJ software version 1.54 (http://imagej.net/ij/). The Hoechst counterstain was used for normalization.

### Lipid peroxidation (MDA) assay

Lipid peroxidation was analyzed by the quantification of Malondialdehyde (MDA) in total protein lysates. Cells were plated in 6-well plates (250,000 cells/well) and treated with 45S5-BG (0.5 mg/ml) or Erastin (1 µM) for 24 h. Cells were then lysed using RIPA lysis buffer supplemented with 0.5% antioxidant supplied with the kit. Protein concentrations were quantified using a BCA protein Assay kit (Thermo Fisher Scientific) before MDA was detected according to the manufacturers protocol. The fluorescent end-product was detected at Ex/Em = 532/553 nm in a FluoStar microplate reader (BMG LabTech). Data were normalized to the protein content of the corresponding sample.

### Statistical analysis

Descriptive statistics, such as mean and standard deviation, were calculated using SPSS software (Version 25; IBM, Armonk, NY, USA). The nonparametric Mann–Whitney *U* test or the Kruskal–Wallis Test were used to compare experimental groups, depending on the number of groups. *P* < 0.05 were regarded as statistically significant and *p* < 0.01 as highly significant. Reported *p*-values are two-sided. Data are presented as mean ± standard deviation. The given number of n always refers to the number of biological replicates. Experiments were done at least in triplicates.

## Supplementary information

Supplemental Figure 1

Legend to supplemental figures 1

Original data

## Data Availability

The data supporting the findings of this study are available from the corresponding author upon reasonable request.
