# Supplementary figures and images for: The mechanism of 45S5 bioactive glass-mediated, cell-type-specific death of bone tumor cells
Source: Cell Death Discov. 2026 Jul 1;12:290. doi: 10.1038/s41420-026-03211-x (PMC13324152; doi:10.1038/s41420-026-03211-x)

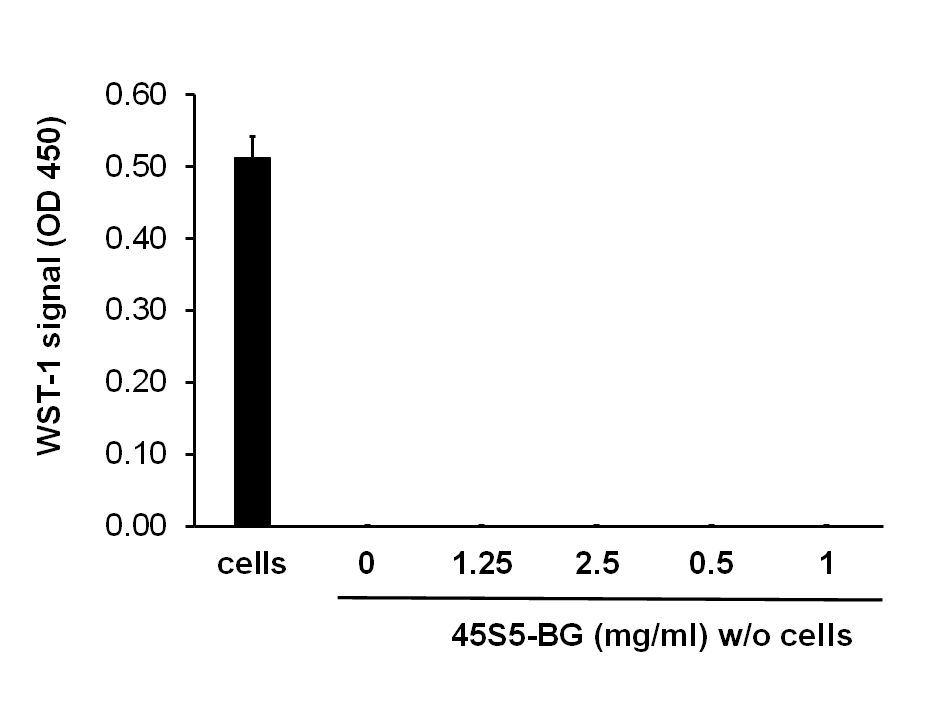

Supplement: Supplementary file 1 — Supplemental Figure 1 [file 41420_2026_3211_MOESM1_ESM.tif]
