# Supplementary material for: The mechanism of 45S5 bioactive glass-mediated, cell-type-specific death of bone tumor cells
Source: Cell Death Discov. 2026 Jul 1;12:290. doi: 10.1038/s41420-026-03211-x (PMC13324152; doi:10.1038/s41420-026-03211-x)
Supplement: Supplementary file 2 — Legend to supplemental figures 1 [file 41420_2026_3211_MOESM2_ESM.docx]

**Supplemental Figure 1.** 45S5-BG particles do not interfere with the WST-1 assay. Different amounts of 45S5-BG particles were subjected to a WST-1 assay without the addition of cells (n=5). HOS143B cells served as positive control. Absorbance values at 450nm are shown.
