# Supplementary material for: The mechanism of 45S5 bioactive glass-mediated, cell-type-specific death of bone tumor cells
Source: Cell Death Discov. 2026 Jul 1;12:290. doi: 10.1038/s41420-026-03211-x (PMC13324152; doi:10.1038/s41420-026-03211-x)
Supplement: Supplementary file 3 — Original data [file 41420_2026_3211_MOESM3_ESM.docx]

**Uncropped Western Blots:**

**Figure 1B:**

**
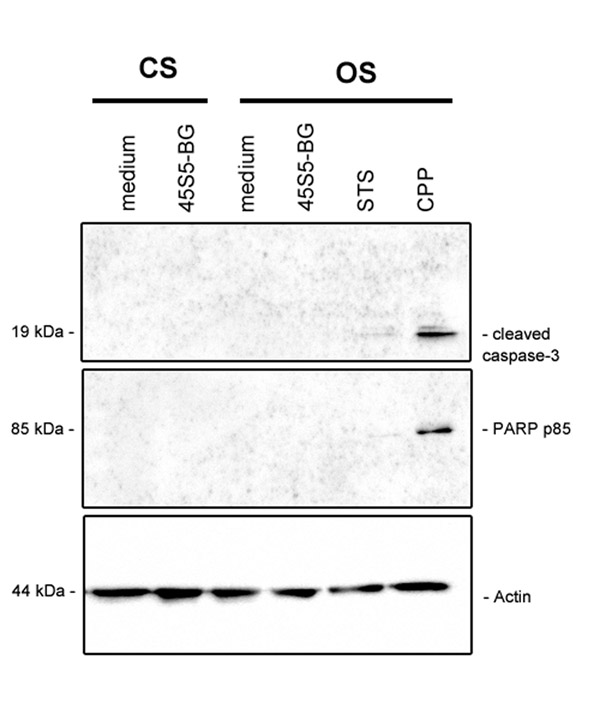
**

**Figure 2B:**

**
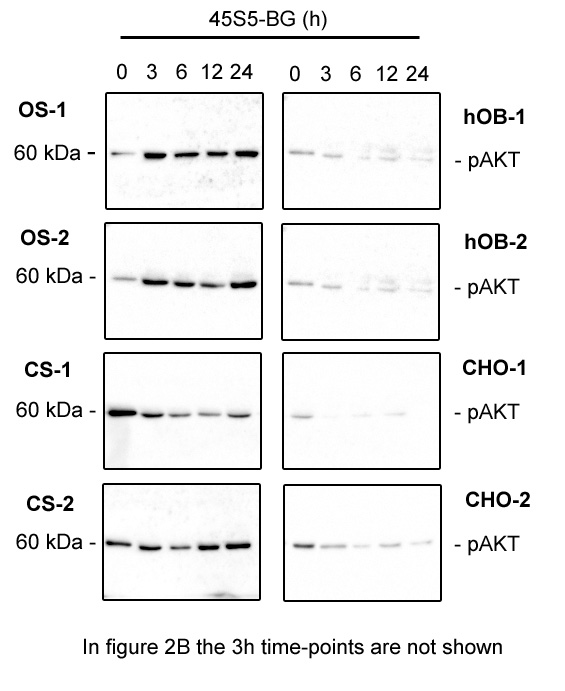
**

**
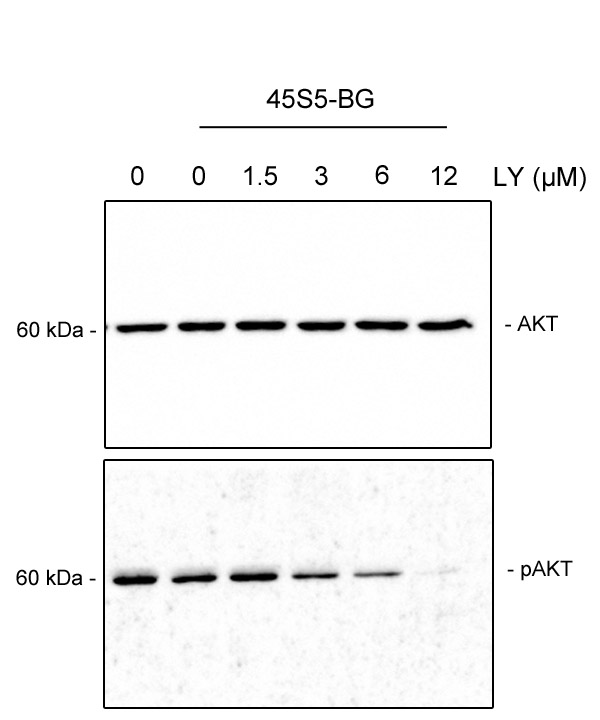
Figure 2D:**

**Figure 3B:**

**
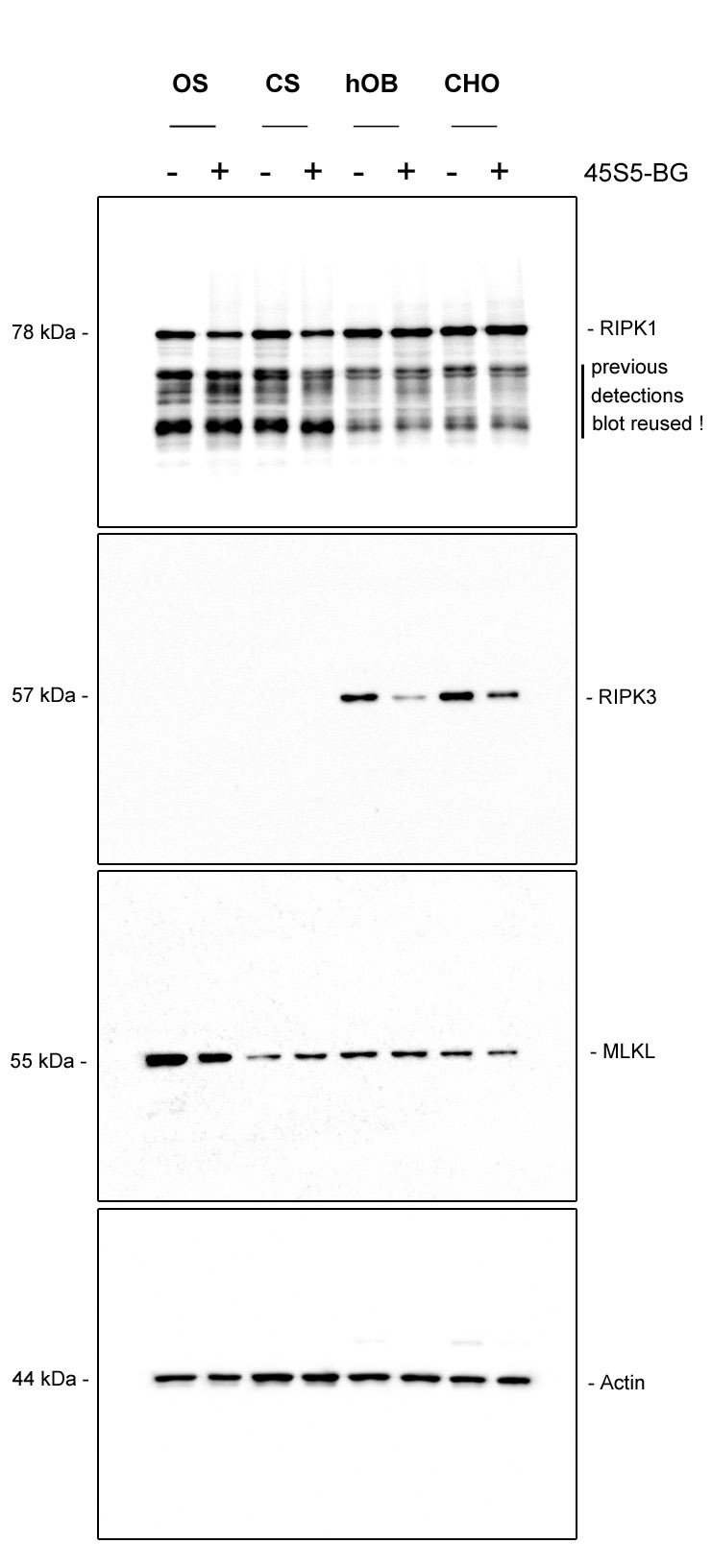
**

**Figure 3B phosphorylated antibodies:**

**
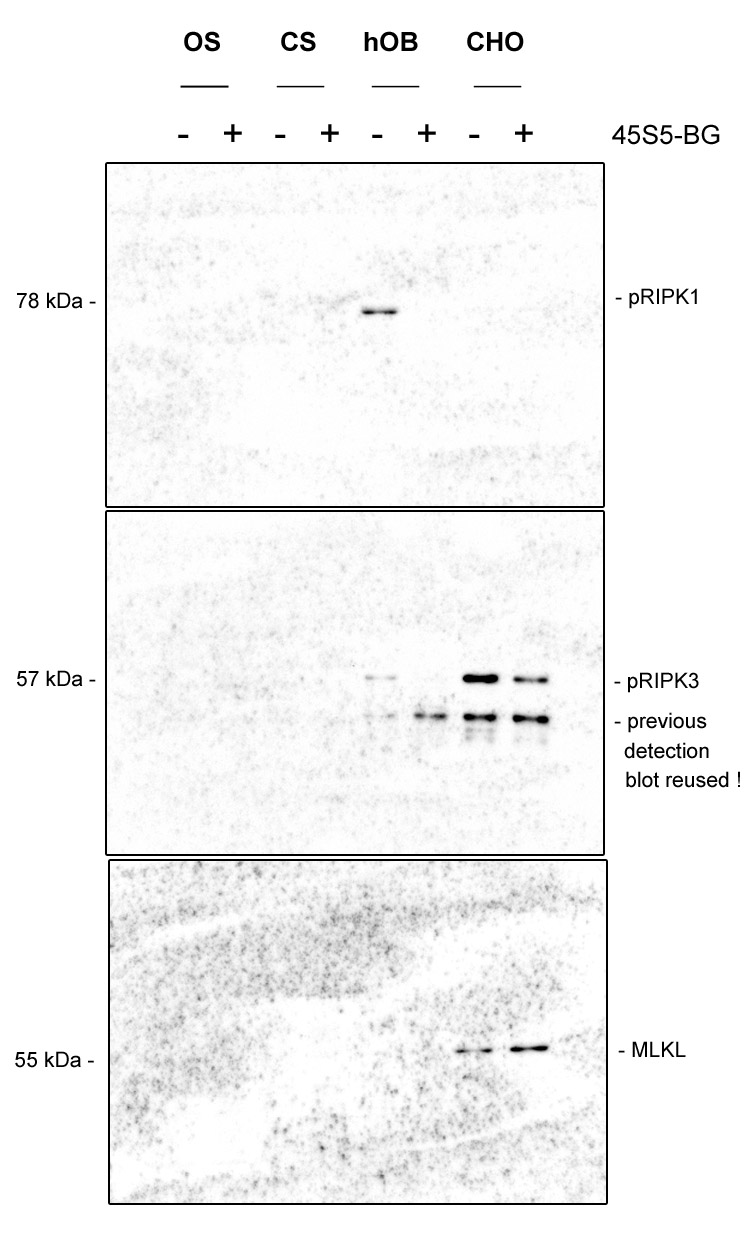
**

**Figure 4A: phospho-p38**

**
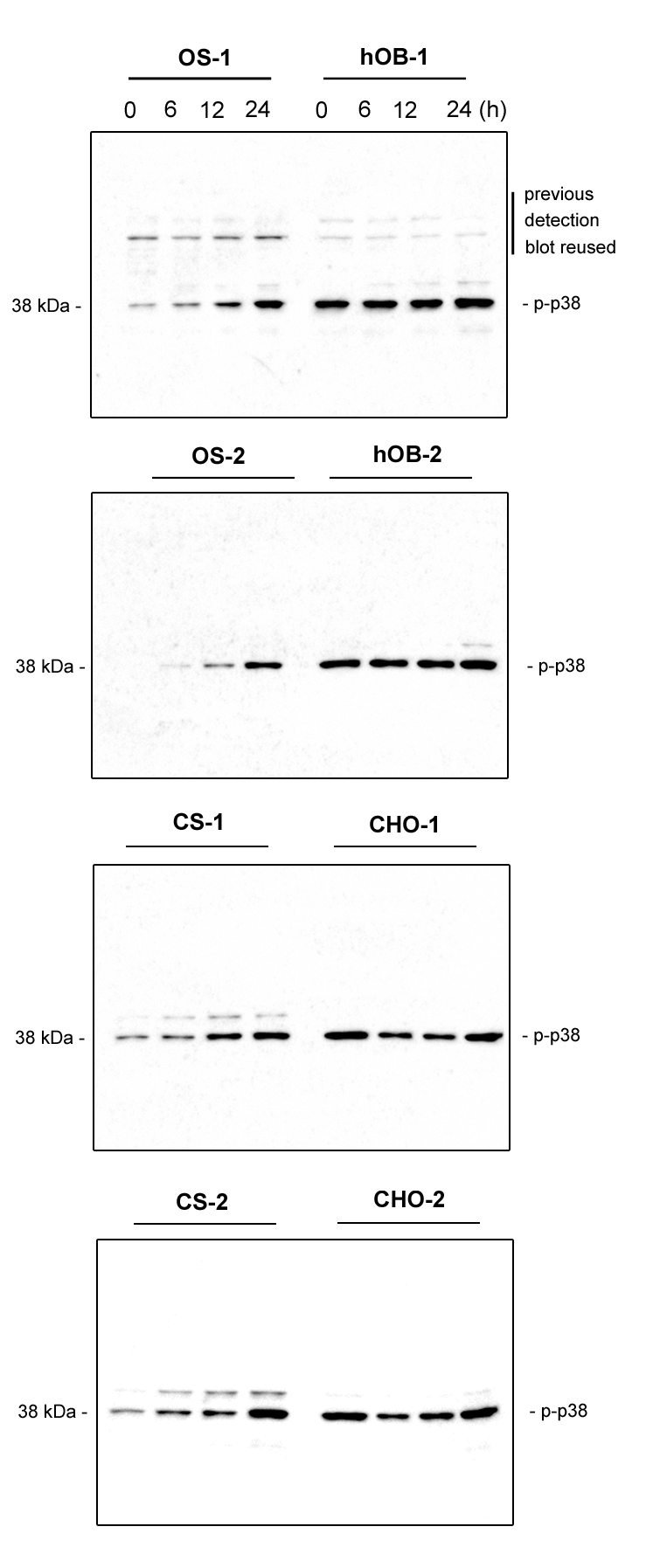
**

**Figure 4A: phospho-JNK**

**
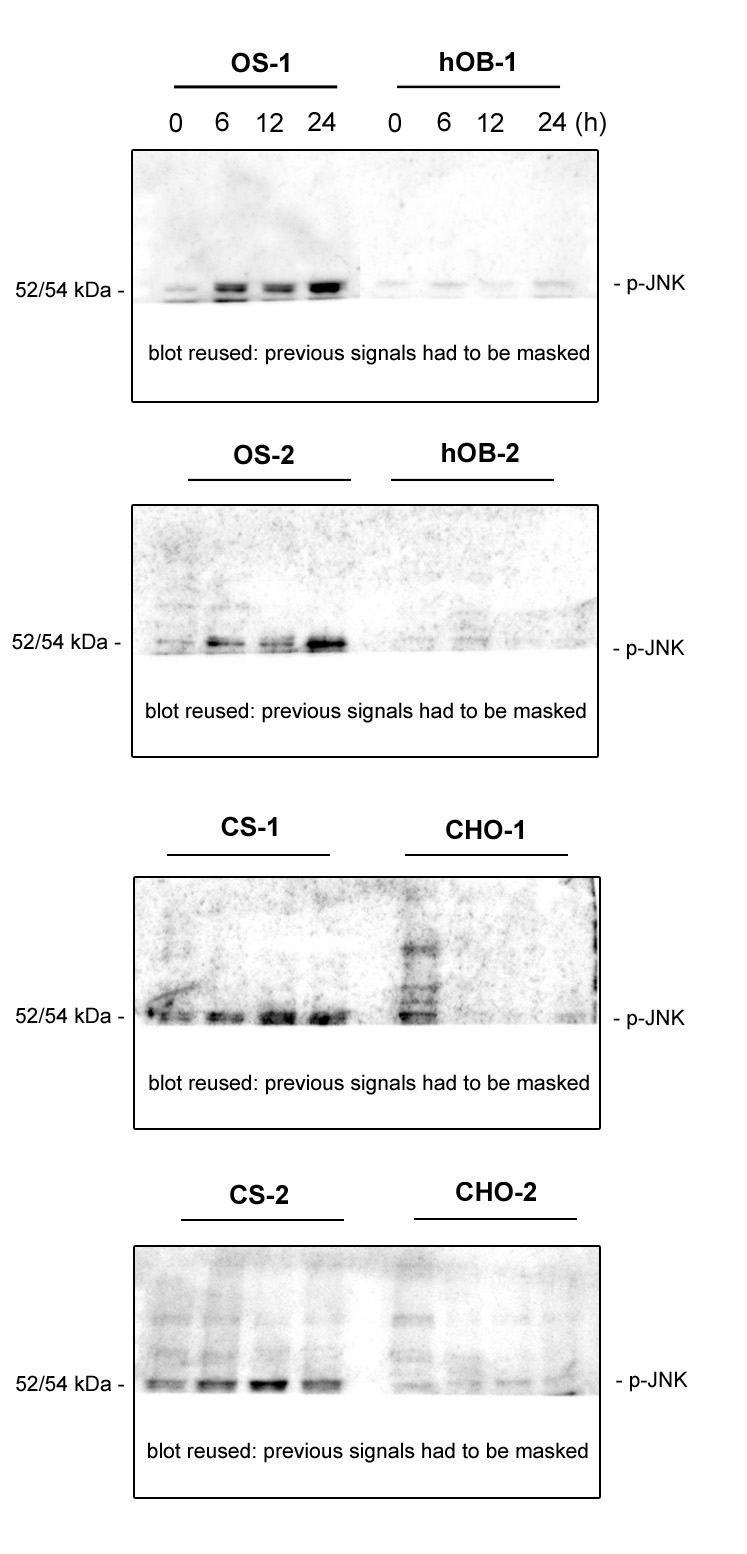
**

**Figure 4A: phospho-ERK1/2**

**
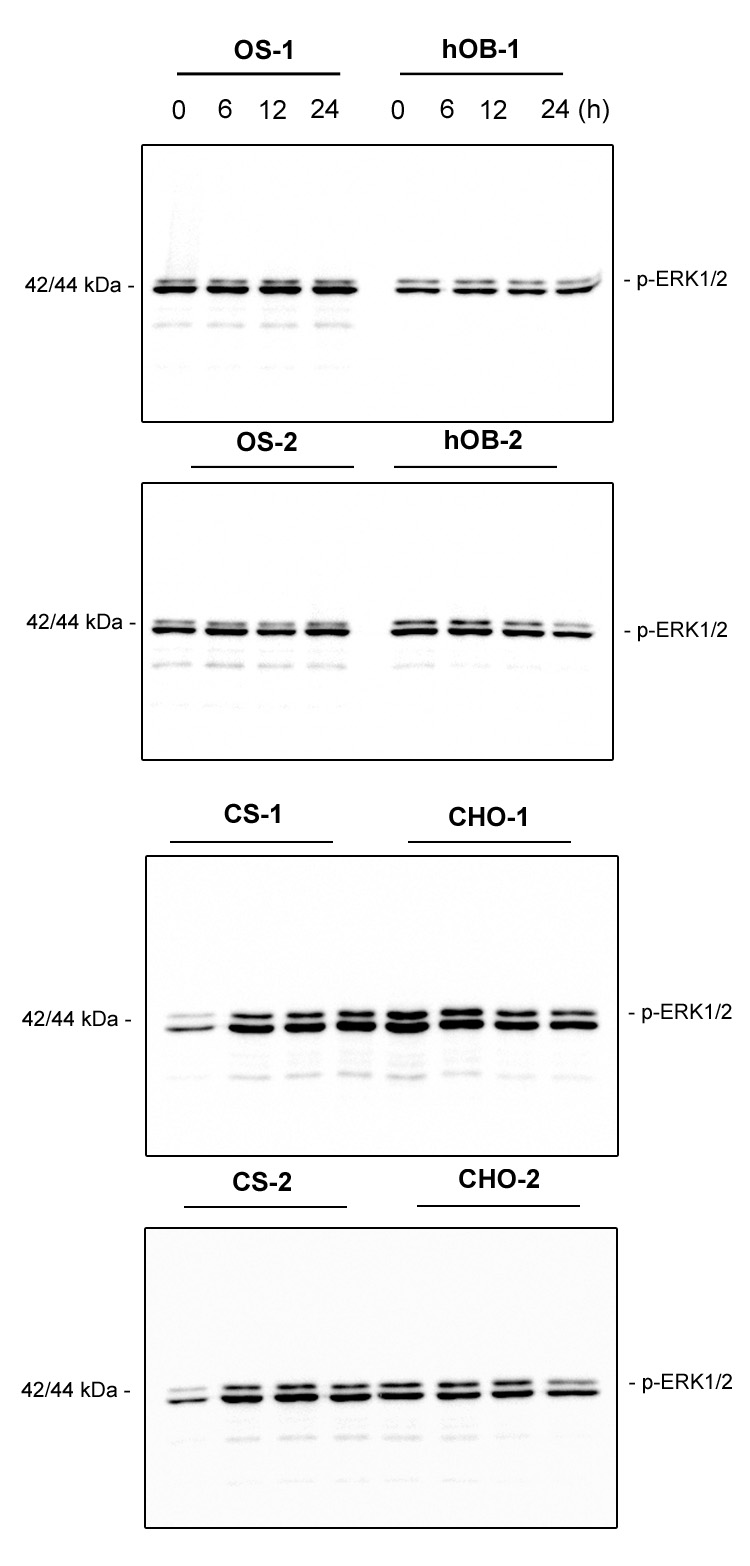
**

**Figure 4A: Actin**

**
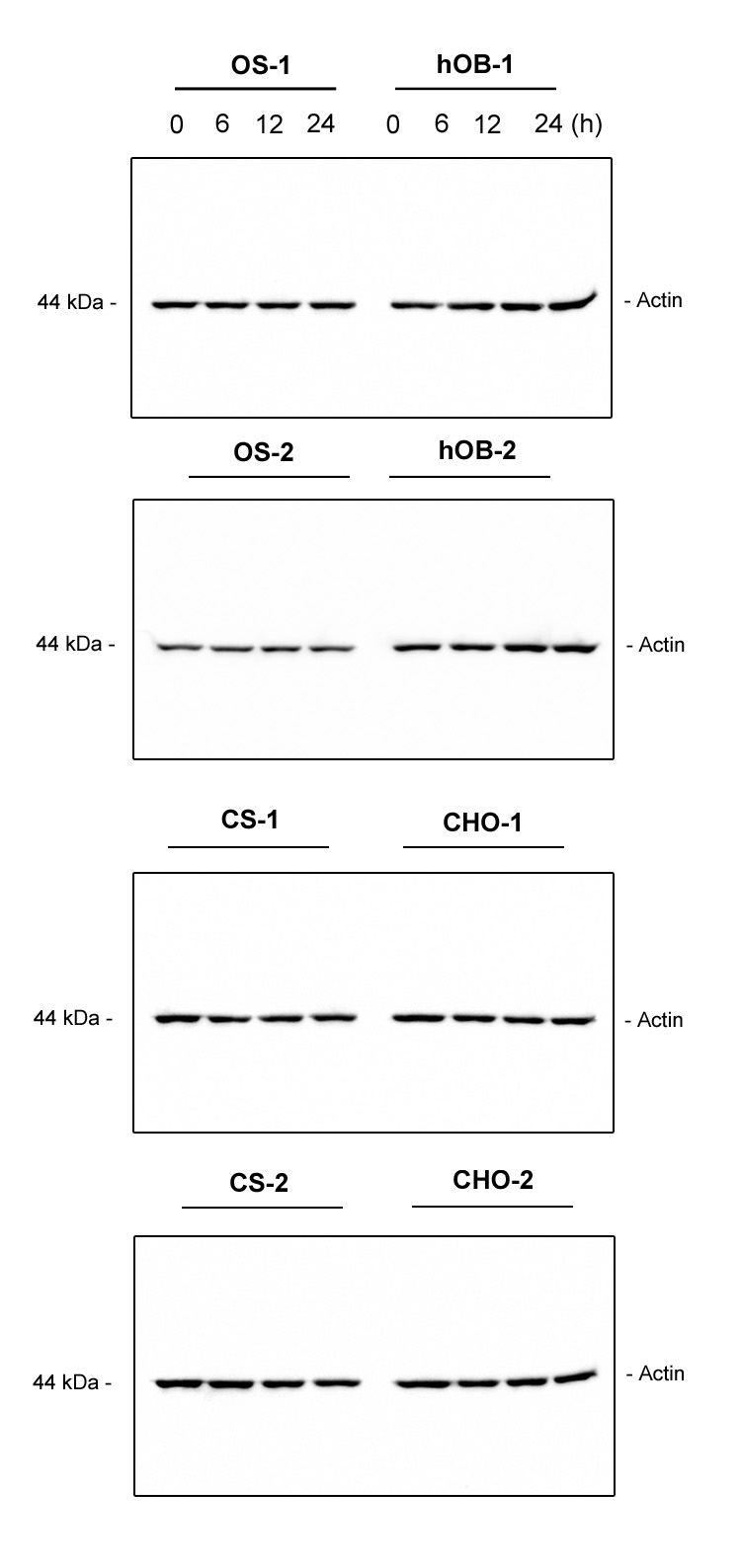
**

**Figure 8E: TfR1 - Actin**

**
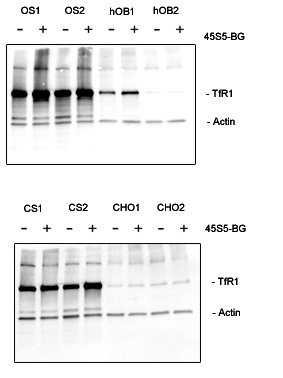
**
